# Aligned Ionogel Electrolytes for High‐Temperature Supercapacitors

**DOI:** 10.1002/advs.201801337

**Published:** 2019-01-22

**Authors:** Xinhua Liu, Oluwadamilola O. Taiwo, Chengyao Yin, Mengzheng Ouyang, Ridwanur Chowdhury, Baofeng Wang, Huizhi Wang, Billy Wu, Nigel P. Brandon, Qigang Wang, Samuel J. Cooper

**Affiliations:** ^1^ School of Chemical Science and Engineering Tongji University Shanghai 200092 P. R. China; ^2^ Dyson School of Design Engineering Imperial College London South Kensington London SW7 2AZ UK; ^3^ Earth Science and Engineering Imperial College London South Kensington London SW7 2AZ UK; ^4^ College of Environmental and Chemical Engineering Shanghai University of Electric Power Shanghai 200090 P. R. China; ^5^ Mechanical Engineering Imperial College London South Kensington London SW7 2AZ UK

**Keywords:** aligned ionogels, supercapacitors, thermal tolerance, tortuosity factors, X‐ray tomography

## Abstract

Ionogels are a new class of promising materials for use in all‐solid‐state energy storage devices in which they can function as an integrated separator and electrolyte. However, their performance is limited by the presence of a crosslinking polymer, which is needed to improve the mechanical properties, but compromises their ionic conductivity. Here, directional freezing is used followed by a solvent replacement method to prepare aligned nanocomposite ionogels which exhibit enhanced ionic conductivity, good mechanical strength, and thermal stability simultaneously. The aligned ionogel based supercapacitor achieves a 29% higher specific capacitance (176 F g^−1^ at 25 °C and 1 A g^−1^) than an equivalent nonaligned form. Notably, this thermally stable aligned ionogel has a high ionic conductivity of 22.1 mS cm^−1^ and achieves a high specific capacitance of 167 F g^−1^ at 10 A g^−1^ and 200 °C. Furthermore, the diffusion simulations conducted on 3D reconstructed tomography images are employed to explain the improved conductivity in the relevant direction of the aligned structure compared to the nonaligned. This work demonstrates the synthesis, analysis, and use of aligned ionogels as supercapacitor separators and electrolytes, representing a promising direction for the development of wearable electronics coupled with image based process and simulations.

Ionogels are gaining increasing interest in the field of flexible electronics due to their wide electrochemical window, good mechanical properties, and thermal stability,^1^ with applications including: lithium batteries,^2^ supercapacitors,^3^ pressure sensors,^4^ fuel cells,^5^ dye‐sensitized solar cells,^6^ actuators,^7^ and soft robots.^8^ In particular, ionogels with ordered structures have recently received attention due to their enhanced ionic transport properties.^9^ Self‐assembly with chemical cross‐linking and phase segregation has been explored as effective means to produce ionogels with ordered micro‐ and nanostructures through the mixing of organic molecules and liquid crystalline polymers. Recently, Liu et al. also reported a method which uses polyethylene glycol (PEG) derivatives as a directional template for the formation of aligned porous structures. The resulting material exhibited anisotropic properties, with improved species transport in a particular direction compared to an equivalent nonaligned ionogel.^10^ However, their applications have been limited by poor thermal and mechanical stabilities resulting in decomposition at high temperatures.^11^ Therefore, there is a need to develop ionogels with improved thermal and mechanical stability, while maintaining good electrochemical performance through structural alignment. However, it remains a great challenge to achieve such targets.

Efforts have been taken to develop functional hydrogels benefiting from aligned structures. Liu et al. who recently reported the preparation of hydrogels with aligned nanosheets containing charged inorganic structures that align cofacially in a magnetic flux, indicating the unique functions of aligned structures.^12^ Additionally, ice‐templating has also been utilized through the directional freezing of water.^13^ In general, ice‐templating is a comparatively convenient method for producing well‐aligned structures when compared to chemical etching and additive manufacturing approaches. Inspired by the ice‐templating method, there is also great potential in converting aligned hydrogels into high‐performance aligned ionogels via solvent replacement techniques. More importantly, there is also a need to understand the root cause of these unique enhancements in order to design better future materials. Exploring the existing research results, scanning electron microscopy (SEM) has been widely used to capture high‐resolution images of the surface of materials and thus enabled a qualitative analysis of the microstructural alignment. However, X‐ray computed tomography (XCT), which has previously been used to nondestructively analyze electrodes^14^ and separators,^15^ enabled the aligned microstructure to be quantitatively analyzed in 3D. The acquired 3D image datasets can then be used to extract a variety of metrics relevant to electrochemical performance, including volume fractions, surface areas, and effective transport properties (such as tortuosity factors).^16^

In this work, we present aligned ionogel via self‐initiated cryopolymerization and later solvent replacement. A precursor solution was prepared by mixing TiO_2_ nanoparticles (TiO_2_‐NPs), clay nanosheets (clay‐Ns), N,N‐dimethylacrylamide (DMAA), and water with the mass ratio of 1:1:0.005:18. As shown in **Figure** **1**a, the positively charged TiO_2_‐NPs can be electrostatically assembled with the negatively charged clay‐NPs to form TiO_2_‐clay composites in the precursor solution. Meanwhile, the DMAA monomer molecules can easily combine with the assembled composites. The aqueous precursor was then directionally frozen with liquid nitrogen at a growth rate of 1 mm min^−1^ with the help of a compressive tester (Figure 1b,c). Here, TiO_2_‐NPs were used not only as the photoinitiator in the cryopolymerization process (by generating valence‐band holes and hydroxyl radicals), but also as a nanoparticle reinforcement to enhance the mechanical strength of the hydrogel via supramolecular effects.^17^ Finally, an aligned ionogel was obtained via ultraviolet (UV) cryopolymerization of the solid precursor for 1.5 h at −18 °C (Figure 1d). To avoid any possible heating effects from the cryopolymerization process, a cold UV‐light source (with an average intensity of 99.8 mW cm^−2^ at 365 nm) was used. For comparison, the precursor was also frozen without directional methods and similarly cryopolymerized at −18 °C, resulting in a more isotropic microstructure. Finally, the obtained hydrogels were freeze‐dried in order to remove the ice directly without damaging the polymer matrixes. A good alignment can be found in the freeze‐dried polymer matrix as shown in Figure 1e. Then the polymer matrixes were soaked in ionic liquid, thus the obtained aligned hydrogel can convert to aligned ionogel via solvent replacement (Figure 1f).

**Figure 1:**
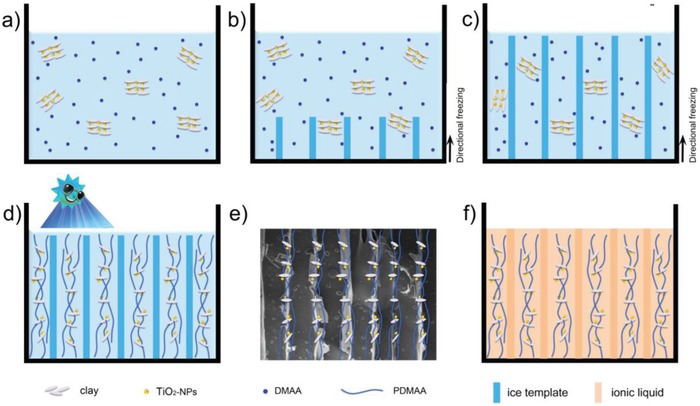
Proposed schematic illustration of preparation of aligned hydrogel applying a directional freezing process. a) The precursor aqueous solution of clay‐Ns, DMAA, and TiO_2_‐NP in water. b,c) The monomer‐nanoparticle composite is excluded from the directionally freezing of the orientated ice crystals. d) The monomer is cryopolymerized to produce hydrogel with an aligned structure. e) SEM image of the freeze‐dried polymer matrix with aligned structure. f) The aligned ionogel can be obtained by adding ionic liquid to freeze‐dried aligned polymer matrix.

**Figure** **2**a,b shows SEM images of the porous microstructures of the nonaligned and aligned gels. The effect of the directional freezing process is clear when comparing the two structures. 1‐Butyl‐3‐methyl imidazolium terafluoroborate (BMIMBF_4_) was selected as the ionic liquid electrolyte for the solvent replacement process due to its high ionic conductivity (3–5 mS cm^−1^ at room temperature), thermal stability, and good wettability.^18^ As shown in Figure S1 (Supporting Information), the size of the sliced freeze‐dried gel matrix is 20 mm in diameter and 0.8 mm in thickness, with the resulting gel having an aligned microstructure throughout the entire thickness. These gel matrices were soaked in BMIMBF_4_ for 24 h, thus ionogels with aligned/nonaligned pore structures were obtained. Figure S2 (Supporting Information) presents frequency sweep curves, showing the rheological properties of the aligned and nonaligned ionogel. Here, both samples show substantial gel‐like elastic responses (*G*′ >*G*″). As shown in Figure S3 (Supporting Information), both the nonaligned and aligned ionogels show relatively good mechanical strength in compressive tests. Finally, carbon nanocages (CNC700) were used as supercapacitor electrode materials because of their high specific surface area (1810 m^2^ g^−1^) leading to enhanced double‐layer capacitance.^19^ The SEM and transmission electron microscopy (TEM) images of the carbon nanocages are shown in Figure S4 (Supporting Information). The resulting supercapacitors were assembled with carbon nanocage electrodes, either side of an integrated ionogel electrolyte. The galvanostatic charge and discharge curves of the aligned and nonaligned ionogel based supercapacitors are shown in Figure 2c with an operating voltage window of 0–3 V at a current density of 1 A g^−1^. Here, the non‐ionogel based supercapacitor achieved a high specific capacitance (*C*_sp_) value of 136 F g^−1^, while the aligned supercapacitor exhibited a 29% higher *C*_sp_ of 176 F g^−1^. Here, the energy efficiency of the aligned supercapacitor is higher (81%) than that of nonaligned supercapacitor (43%), indicating the aligned architecture significantly reduces losses in the system, thus contributing to an improved charge/discharge performance.^20^ Furthermore, the supercapacitors were discharged at various current densities. As shown in Figure 2d, at low current densities (0.5 A g^−1^), the specific capacitance of both ionogel systems was approximately equal (180 F g^−1^) with the *C*_sp_ decreasing monotonically with the increasing current densities. However, for all current densities above 0.5 A g^−1^, the aligned ionogel based supercapacitor showed significantly better performance and at 5 A g^−1^ *C*_sp_ was more than double that of the nonaligned device.

**Figure 2:**
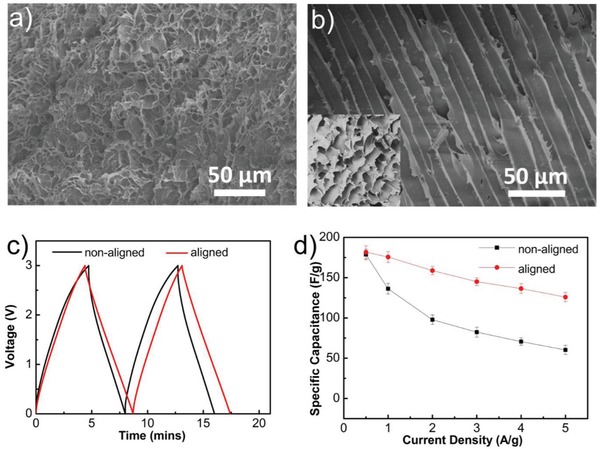
SEM images of the 3D structures of the gels after vacuum freeze‐drying treatment. a) Nonaligned porous polymer structure of the gel matrix without directional freezing treatment; b) SEM images from longitudinal and cross sections of the aligned porous polymer structures (inset image) of the gel matrix applying directional freezing process. c) Galvanostatic charge–discharge curves of the nonaligned and aligned ionogel electrolyte based supercapacitors measured at 1 A g^−1^. d) The specific capacitance of the two ionogels at various current densities for the supercapacitors.

Mercury (Hg) intrusion was performed to determine the distribution of open pore sizes for the two samples (Figure S5, Supporting Information). The polymer matrix of the aligned ionogel exhibits a high open porosity of 91% with the majority of pores between 0.2 and 10 µm, while the nonaligned ionogel has a lower open porosity of 54% with a similar pore size distribution. However, mercury intrusion does not allow for direct quantification of the nature of the transport properties, nor is it able to investigate closed pores. As both the solid and pore phases of the ionogel are expected to participate in the ion transport, the closed pores will be important to consider. To address this issue, the 3D microstructures of the aligned and nonaligned ionogel samples were captured using a laboratory XCT instrument (nanotom s, GE Sensing and Inspection Technologies GmbH, Wunstorf, Germany) operated at an X‐ray tube voltage and current of 80 kV and 225 µA, respectively, with a tungsten‐on‐diamond target. Postprocessing and segmentation of the reconstructed 3D datasets^21^ was performed using the Avizo software package (v 9.4., Thermo Fisher Scientific, USA).

The reconstructed 3D data were then segmented using the open‐source Trainable Weka Segmentation (TWS) tool, which is a plugin available as part of the Fiji distribution of ImageJ.^22^ TWS is a convolution‐based segmentation tool which is iteratively trained based on user‐generated labels. The initial grayscale image, segmentation overlay, and binarized data for a single slice of each of the two samples can be seen in the first three columns of **Figure** **3**. The porosities of the aligned and nonaligned samples were found to be 60 and 80%, respectively. The segmented 3D datasets were then cropped to cuboid regions (834 × 770 × 1277 voxels for the nonaligned structure and 446 × 453 × 513 voxels for the aligned sample) which only contained the sample structures and excluded the surrounding free space. The fourth column of Figure 3 shows a 3D rendering of the cropped, segmented datasets, as well as labeling for the three axis directions. These cropped volumes were then used as the basis of 3D diffusion simulations, performed using the *TauFactor* MatLab application.^23^ TauFactor iteratively calculates the steady‐state diffusive flux resulting from the potential difference between two parallel Dirichlet boundaries. This allows the effective transport properties of the medium to be calculated, as described in the literature.^23^ For comparison of the two ionogel samples, the relative diffusivity will be used, *D* ^rel^, which is a dimensionless ratio of the simulated effective diffusivity, *D* ^eff^, and the intrinsic diffusivity of the material in question, *D*_0_, as described in Equation (1)

(1) $$D^{\mathrm{rel}}=\frac{D^{\mathrm{eff}}}{D_{0}}=\frac{\varepsilon }{\tau }$$

**Figure 3:**
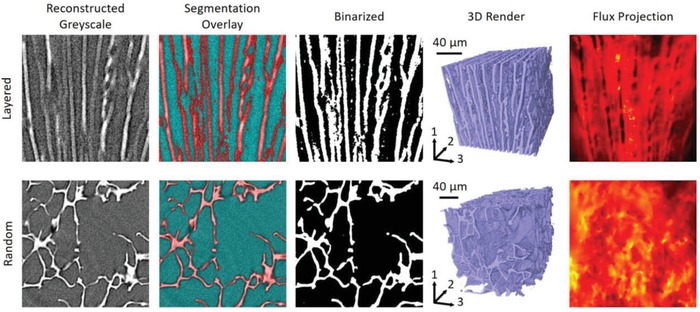
The imaging and simulation pipeline for the two samples, showing the reconstructed grayscale images from the XCT; the same images, overlaid with the trained Weka segmentation results; the same images, now converted to binary format; the full segmented 3D volume (showing scale and directional labeling); and, finally, sample simulation results highlighting the projected flux density resulting from the diffusion simulation, where the bright regions represent higher flux.

This simplified approach assumes that transport only occurs in the pores and so *D*_0_ represents the diffusivity of ions in the bulk electrolyte. As such, ε is the volume fraction of the pores in the system (i.e., the porosity) and τ meets the conventional definition of the tortuosity factor.

Although the principal transport paths for the ions in these ionogel structures are through the pores, it has been established in the literature^24^ that the ionogel itself is a good ionic conductor. As such, two sets of simulations were conducted in *TauFactor* to investigate this effect. In the first case, the simulation was “pores only” as described above, but the second set of simulations also allowed transport through the ionogel phase by attributing a diffusivity of *D*_ionogel_ = 0.01 × *D*_0_. This estimate was based on literature values for similar materials.^25^ Equation (2) can be used to calculate the relative diffusivity of the “two‐phase” simulation, where, once again, *D* ^rel^ gives the diffusivity compared to that of the electrolyte alone. The summation term uses the index *i* to count through the phases (in this case there are only 2, but the approach generalizes). As expected, if *D* is set to zero for one of the phases, Equation (2) simplifies to Equation (1)

(2) $$D^{\mathrm{rel}}=\frac{D^{\mathrm{eff}}}{D_{0}}=\frac{\sum \frac{D_{i}}{D_{0}}\varepsilon_{i}}{\tau }$$

The results of these simulations are summarized in **Table** **1**, where it can be seen that the nonaligned structure has fairly isotropic transport properties, which was in line with expectations. The layered structure, however, is highly anisotropic, with diffusivity varying by an order of magnitude depending on the direction considered. This result is observed irrespective of whether the ionogel participates in the transport process, although the effect is more pronounced in the pores‐only approach.

**Table 1:** Relative diffusivities calculated using *TauFactor* for the two samples in the three directions as described in Figure 3

		Directional relative diffusivities		
		1	2	3
Pore only	Random	0.31	0.33	0.29
	Layered	0.48	0.29	0.02
Two phase	Random	0.39	0.40	0.38
	Layered	0.51	0.35	0.07

Interestingly, the aligned sample, despite having a lower porosity, was found to have the highest relative diffusivity when the appropriate direction was considered. This suggests that the aligned structure is highly beneficial to transport in the through‐plane direction, which is also in line with expectations. To understand why the transport in direction 2 of the aligned structure is comparable to that of the random sample, it is necessary to point out that the layers are held together by struts, which hinder transport in this direction. The final column of Figure 3 shows a map of the local fluxes resulting from the transport simulations of the pores‐only analysis, which have been summed through the volume and then normalized, in order to show them in 2D. These flux maps highlight both the influence of the aligned structure on the transport as well as the presence of transport limiting bottlenecks in the nonaligned sample.

Meanwhile, another significant advantage of ionic liquids and ionogels is their high thermal stability. Figure S6 (Supporting Information) shows the thermal stabilities of the ionic liquid (BMIMBF_4_) and BMIMBF_4_ based aligned ionogel. As shown in the thermal gravimetric analysis (TGA) curves, both ionic liquid and its aligned ionogel possess a high thermal stability with the low mass loss regimes up to an operating high temperature of nearly 400 °C. In view of its impressive thermal stability, the aligned ionogel based supercapacitor was further tested at high temperatures up to 200 °C. **Figure** **4**a presents the cyclic voltammetry (CV) curves of the aligned system, which become increasingly rectangular as the temperature increases from 25 to 200 °C. The correlation of *C*_sp_ with current density at various temperatures is shown in Figure 4b. Here, the accessible *C*_sp_ increases with temperature for the same current density due to increased ionic conductivity. This amounts to an increase in *C*_sp_ of 108% at a current density of 10 A g^−1^ from 80 F g^−1^ at 25 °C to 167 F g^−1^ at 200 °C. Figure 4c presents the ionic conductivities and the viscosities of the aligned ionogel at various temperatures. When the temperature increased from 25 to 200 °C, there is a 530% increase in ionic conductivity from 3.5 to 22.1 mS cm^−1^ and a 87% decrease in viscosity from 13 216 to 1737 Pa s. The temperature‐dependent electrochemical enhancement can be further illustrated in Figure 4e and Figure S7 (Supporting Information), which shows the impedance spectra of the ionogel based supercapacitors measured at various temperatures.

**Figure 4:**
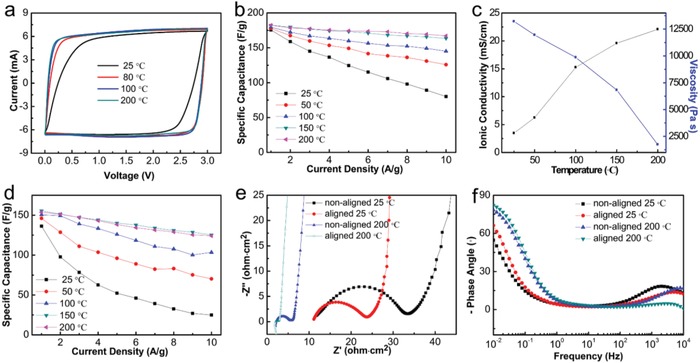
a) CV curves of the aligned ionogel electrolyte based supercapacitor applying various temperatures (25, 80, 100, and 200 °C). b) The correlation of specific capacitance with current density applying various temperatures for aligned ionogel based supercapacitor. c) The effect of temperature on the ionic conductivity and viscosity of the aligned ionogel electrolyte. d) The correlation of specific capacitance with current density applying various temperatures for nonaligned ionogel based supercapacitor. e) Nyquist plots and f) Bode plots of the phase angle versus frequency of the aligned and nonaligned ionogel based supercapacitors applying various temperatures (25 and 200 °C).

Both the aligned and nonaligned supercapacitors achieved better capacitive performance and rate capability at higher temperatures, but the aligned ionogel based supercapacitor consistently achieved superior electrochemical performances at all temperatures relative to the nonaligned device (Figure S8, Supporting Information and Figure 4d). From the Bode phase diagram (Figure 4f), it can be seen that the phase angle is −54.6°, −66.3°, −78.3°, and −82.1° at 10 mHz for the nonaligned 25 °C, aligned 25 °C, nonaligned 200 °C, and nonaligned 200 °C, respectively, which confirms that the aligned supercapacitor, at high temperature, exhibits best capacitive performance, implying it can access the most surface area.

In all cases, the general shape of the impedance spectra matched with the standard response described in the literature for supercapacitors with nanostructured carbon electrodes;^26^ with a real axis intercept at high‐frequency indicating the series resistance, a near vertical section at low‐frequency indicating imperfect capacitive behavior, and an approximately semicircular region between the two. High‐frequency semicircular features are often attributed to pseudocapacitive charge‐transfer processes, such as redox reactions within the electrolyte,^27^ or specific adsorption,^28^ which Eftekhari suggests may be inevitable for nanostructured carbon electrodes. An alternative interpretation is offered by Eloot et al.,^29^ who reproduced this feature simply through the simulation of the spatially distributed capacitance inherent to porous electrodes. It is critical to remember that the only difference between the two supercapacitors measured in this experiment was the morphology of their separators; however, the width of the near semicircular feature for the random sample was approximately double that of the aligned sample at 25 °C, with the difference being even more pronounced at 200 °C. The separator is not referred to in any of the above explanations, which suggests that an alternative interpretation may be required.

Transport through the convoluted two‐phase network of these separators will involve contributions from both migration and diffusion processes. A recent study by the authors simulated the impedance response of diffusive processes in 3D pore networks with boundary conditions analogous to the 1D finite length Warburg (FLW) element. It was found that both the shape and frequency distribution could be made to deviate significantly from the analytical 1D FLW case by modifying the geometry of the transport paths.^30^ Ultimately, this feature may result from a combination several of the described processes that are difficult to deconvolve due to their insufficiently distinct time constants. However, it is clear that the electrochemical performance was significantly improved by the choice of separator morphology of aligned structure.

In conclusion, a solvent replacement method to prepare ionogels with aligned pore structures via self‐initiated cryopolymerization is described which can be used as an integrated electrolyte and separator for high‐temperature supercapacitors. This is a simple and effective approach to prepare aligned ionogels with the combined high mechanical strength, thermal stability, and electrochemical performance necessary for operation in harsh environments. This work highlights the electrochemical performance enhancements of supercapacitors with aligned structures (109% improvement in *C*_sp_ at 5 A g^−1^, 25 °C) and at high temperatures (530% improvement in ionic conductivity from 25 to 200 °C). The microstructure of the aligned and nonaligned ionogel structures investigated here was imaged using XCT and the data were used as the basis for 3D diffusion simulations. The aligned system was highly anisotropic with one order of magnitude difference in diffusive properties between orientations and superior conductivity exhibited in the principal aligned direction compared to the isotropic nonaligned ionogel. This therefore opens new opportunities for the design of novel high‐performance materials for energy storage applications.

## Conflict of Interest

The authors declare no conflict of interest.

## Supporting information

Supplementary

Supplementary

Supplementary
