# Supplementary material for: Aligned Ionogel Electrolytes for High‐Temperature Supercapacitors
Source: Adv Sci (Weinh). 2019 Jan 22;6(5):1801337. doi: 10.1002/advs.201801337 (PMC6402534; doi:10.1002/advs.201801337)
Supplement: Supplementary file 1 — Supplementary [file ADVS-6-1801337-s002.pdf]

Copyright WILEY-VCH Verlag GmbH & Co. KGaA, 69469 Weinheim, Germany, 2018.

ADVANCED SCIENCE
Open Access

Supporting Information

for *Adv. Sci.,* DOI: 10.1002/advs.201801337

Aligned Ionogel Electrolytes for High-Temperature Supercapacitors

Xinhua Liu, Oluwadamilola O. Taiwo, Chengyao Yin, Mengzheng Ouyang, Ridwanur Chowdhury, Baofeng Wang, Huizhi Wang, Billy Wu, Nigel P. Brandon, Qigang Wang, and Samuel J. Cooper*

Copyright WILEY-VCH Verlag GmbH & Co. KGaA, 69469 Weinheim, Germany, 2016.

Supporting Information

Aligned Ionogel Electrolytes for High Temperature Supercapacitors

By Xinhua Liu, Oluwadamilola O. Taiwo, Chengyao Yin, Mengzheng Ouyang, Ridwanur Chowdhury, Baofeng Wang, Huizhi Wang, Billy Wu, Nigel P. Brandon, Qigang Wang* and Samuel J. Cooper

Corresponding Author

*E-mail: wangqg66@tongji.edu.cn.

1. Materials

1-butyl-3-methylimidazolium terafluoroborate ($BMIMBF_4$) were purchased from Lanzhou Institute of Chemical Physics, TiO_2 nanoparticles solution were purchased from Dekedaojin (Beijing) Co., Ltd. N, N-dimethylacrylamide (DMAA), were purchased from Sinopharm Chemical Reagent Co., Ltd (Shanghai, China). All materials and reagents were used without further purification. Acetylene black and polytetrafluoroethylene (PTFE, 60 wt% aqueous dispersion) were obtained from Shanghai 3F New Material (Shanghai) and Aladdin Chemistry Co., Ltd.

2. The preparation of aligned/non-aligned ionogel

The precursor was directionally frozen in liquid Nitrogen at movement rate of 1 mm/min with the help of a tensile-compressive tester. Afterwards, the preparation of the ionogel was realized by UV light polymerization for 1.5 hours at -18 °C refrigerator. The UV lamp was a cold point light-source (Shanghai Huijun Science＆Technology Devel Opment Co., Ltd), which could produce a high intensity of 99.8 mW/cm^2 at 365 nm. The non-aligned hydrogel could be obtained by following the same process but without directional freezing. The as-prepared hydrogels were vacuum freeze-dried and then put in $BMIMBF_4$ for 12 hours. Finally, the aligned/non-aligned ionogel can be prepared.

3. Pre-treatment and characterization

3.1 Pre-treatment of Scanning Electron Microscope (SEM) samples

The as-prepared aligned/non-aligned hydrogels were dried via vacuum-freeze drying (Freezing Dryer, Scientz–10N, Ningbo Scientz Biotechnology Co., Ltd.). Then, the above samples were coated with a thin layer of gold by the conductive coating process (with a thickness of about 5 nm). Finally, the polymer structures of the samples were observed by scanning electron microscopy (S-4800, Hitachi High-Technologies Corporation, Tokyo, Japan) at a voltage of 1 kV.

3.2 Characterizations

The compressive measurements of the ionogels were monitored by a tensile-compressive tester (FR-108B, Farui Co.). The ionogel samples were set on the lower plate, after that, the samples were compressed by the upper plate at a speed of 1 mm/min. The rheological experiments were performed on a Thermo Haake Rheostress RS6000 rheometer (Thermo Scientific, Karlsruhe, Germany) with parallel-plate geometry (with diameter of 20 mm).

The reaction mixtures were obtained using different reaction times, and about 5 mg of each sample was put in a hermetically sealed aluminum oxide pan. The experiments were carried out under a nitrogen atmosphere at a heating rate of 10 ºC/min over the temperature range from 30 ºC to 600 ºC.

The pore size and distribution were also determined by Hg porosimetry (AutoPore IV 9500).

Ethanol was used as the displacement liquid because it can easily penetrate into the polymer pores without inducing shrinkage or swelling as a nonsolvent of the polymers.[1] The porosity was measured according to the following equation:

$$\mathrm{porosity}(\%) = \frac{volume\ occupied\ by\ ethanol}{voclume\ of\ polymer + volume\ occupied\ by\ ethanol}$$

3.3 X-ray Tomography

The analysed samples were positioned between the X-ray source and detector to provide an effective pixel size of 0.9 μm. The 3D structures were reconstructed using 2,400 projection images captured over a 360° sample rotation and a cone-beam filtered back projection algorithm based on the Feldkamp-Davis-Kress (FDK) algorithm. Each projection image was acquired with an X-ray detector integration time of 2 s, with averaging and skip settings of 3 and 1 respectively, and an activated detector shift to minimise ring artefacts.

4. Electrochemical properties of ionogel electrolytes

Cyclic voltammetry, alternating current impedance sectroscopy, and chronopotentiometry were

monitored by a Metrohm Autolab PGSTA302N potentiostat-galvanostat (The Netherlands). Carbon nanocages were prepared and used as electrode materials. The electrodes were prepared with the mixture of carbon nanocage powder, Polytetrafluoroethylene (PTFE), and acetylene black with a w/w ratio of 75:15:10. The electrodes were dried in vacuum at 110 °C for 12 h. Titanium plated stainless steel wire (500 mesh) was selected as current collector. Finally, the electric double layer supercapacitor was fabricated using two electrodes and an ionogel electrolyte. The supercapacitors were fabricated in button batteries with a force of about 0.2 N, cross-sectional area 2 cm^2, and an ionogel thicknessof 1000 um. The aligned ionogel was vertically put in supercapacitor as follows.

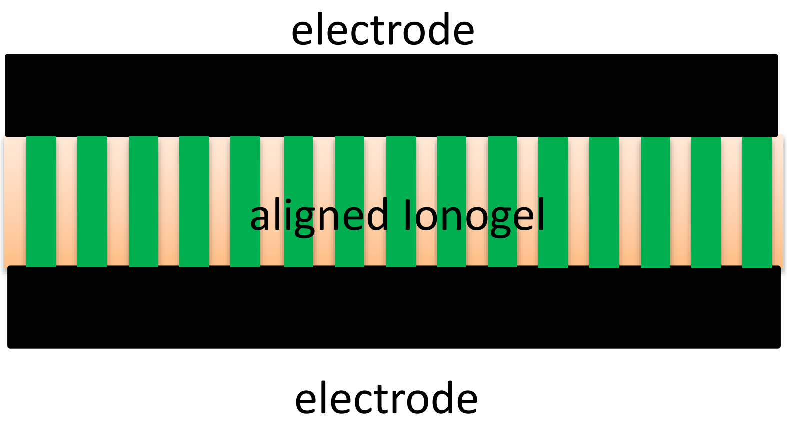

AC impedance measurements were employed to measure ionic conductivity of the ionogels. The ionogel electrolytes were SS/electrolyte/SS (stainless steel electrode: SS) sandwiched structured coupled with a polytetrafluoroethylene (PTFE) spacer which can provide a circular hole to hold the ionogel electrolyte. The AC impendence tests were measured over the frequency range of 100 kHz to 0.1 Hz. The conductivity of the electrolyte is calculated according to the Equation:

$\sigma = l/(AR_b)$

where R_b is the resistance of the bulk electrolyte, l is the thickness of the gel and A is the area of electrode covered by gel.[2]

5. Figures

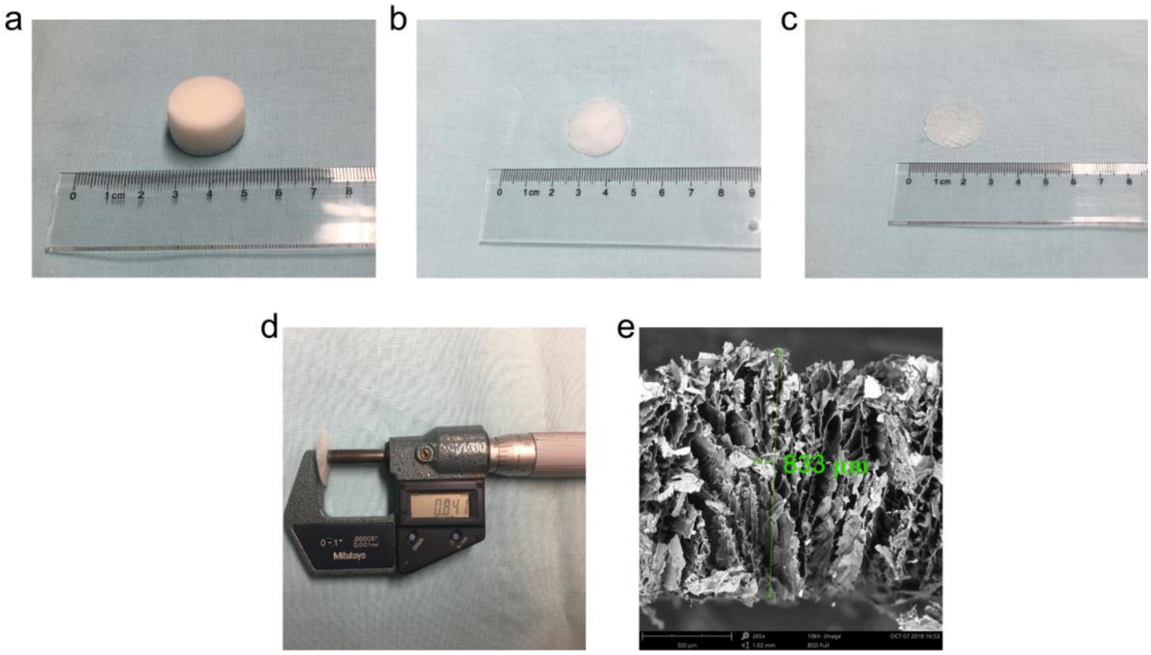

Figure S1. a) Photograph of the freeze-dried polymer matrix with aligned structure. Photographs of b) sliced polymer matrix film and c) after adding ionic liquid. d) Thickness of sliced polymer matrix film (thickness: 0.841 mm) and e) SEM side view of the aligned polymer matrix (thickness: 0.833 mm).

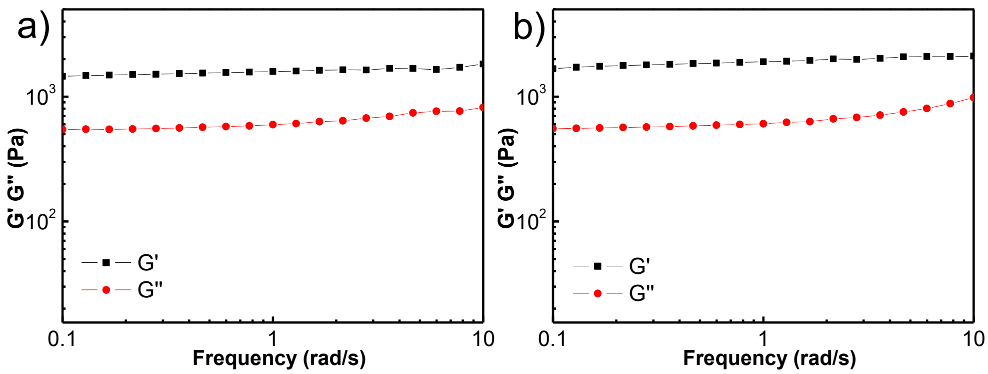

Figure S2. The frequency sweep curves of the $BMIMBF_4$-based non-aligned ionogel and aligned ionogel at a fixed strain of 1%, applying a frequency range of 0.1 -10 Hz

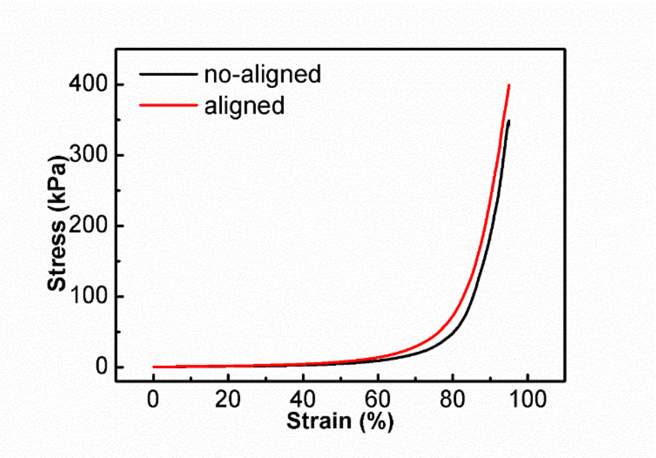

Figure S3. Compressive curves of the $BMIMBF_4$-based non-aligned ionogel and aligned ionogel.

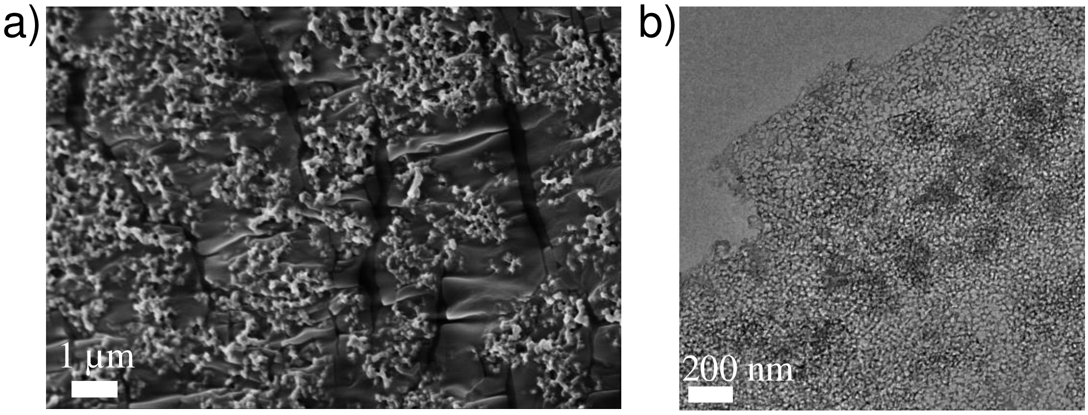

Figure S4. a) SEM image of the carbon nanocages. b) TEM image of the carbon nanocages.

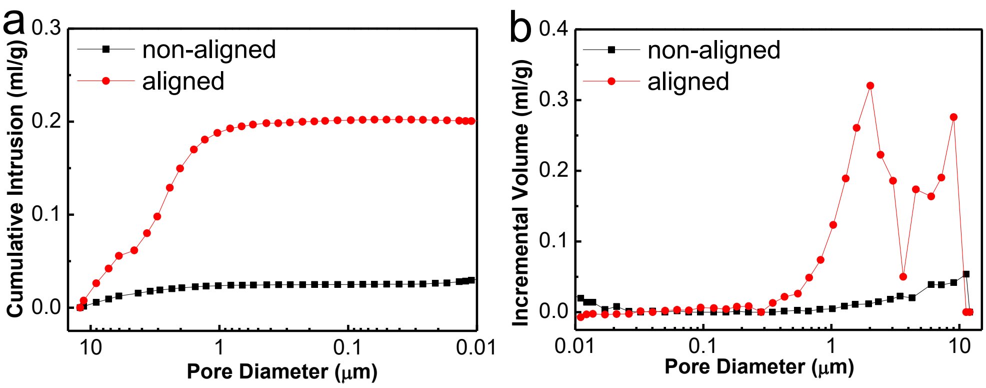

Figure S5. Pore size distribution of the aligned and non-aligned polymer matrixes. (a) Hg cumulative intrusion, and (b) Hg incremental volume versus pore diameter of the aligned and non-aligned samples.

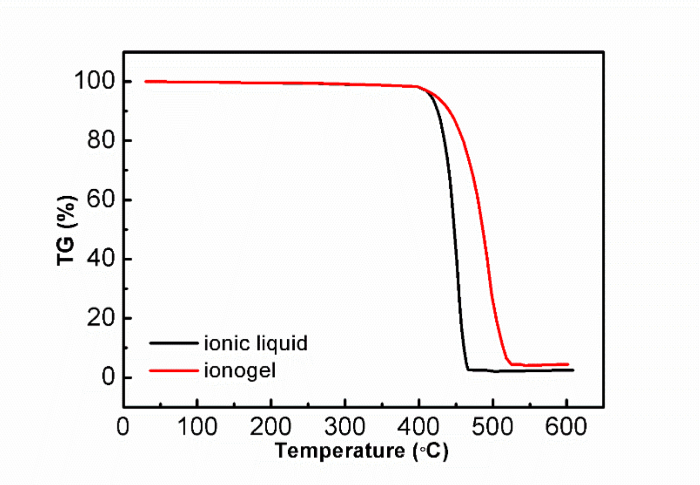

Figure S6. TG curves of (a) the ionic liquid ($BMIMBF_4$) and (b) $BMIMBF_4$-based aligned ionogel at the temperature range of 30 °C to 600 °C at a heating rate of 10 °C min^{-1}.

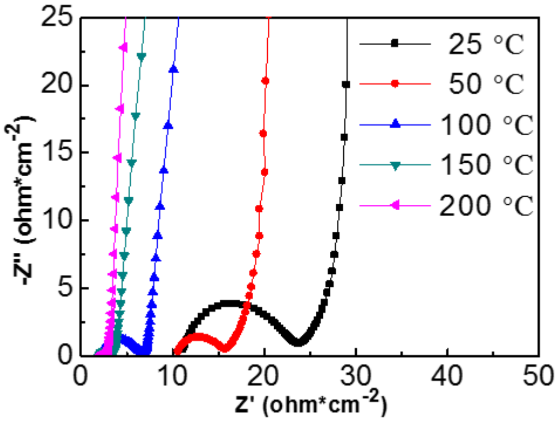

Figure S7. The impedance of the aligned ionogel based supercapacitor at various temperatures.

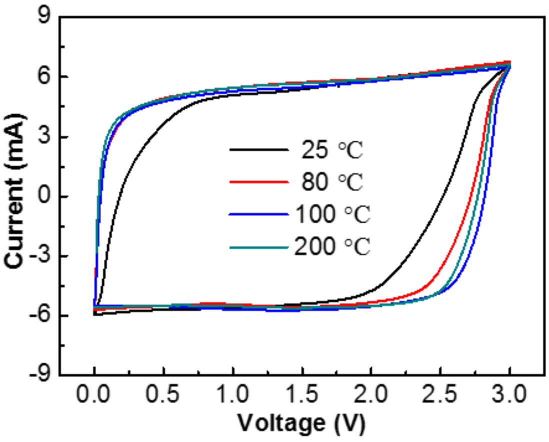

Figure S8. CV curves of the non-aligned ionogel electrolyte based all-solid-state supercapacitor applying various temperatures (25 ℃，80 ℃, 100 ℃, and 200 ℃).

6. References

[1] R. Zhang, P. X. Ma. J Biomed Mater Res, 1999, 44, 446－455

[2] a) J. Malathi, M. Kumaravadivel, G. Brahmanandhan, M. Hema, R. Baskaran, S. Selvasekarapandian, J. Non-Cryst. Solids **2010**, 356, 2277; b) S. Rajendran, O. Mahendran, R. Kannan, J. Phys. Chem. Solids **2002**, 63, 303.
