# Supplementary figures and images for: Aligned Ionogel Electrolytes for High‐Temperature Supercapacitors
Source: Adv Sci (Weinh). 2019 Jan 22;6(5):1801337. doi: 10.1002/advs.201801337 (PMC6402534; doi:10.1002/advs.201801337)

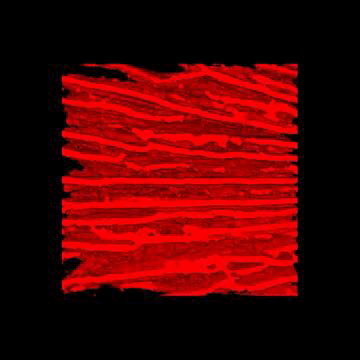

Supplement: Supplementary file 2 — Supplementary [file ADVS-6-1801337-s001.GIF]

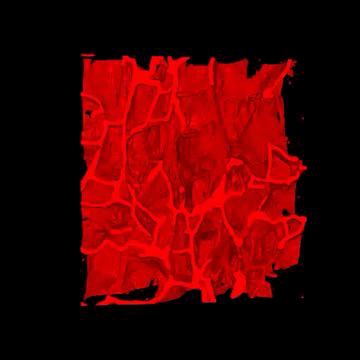

Supplement: Supplementary file 3 — Supplementary [file ADVS-6-1801337-s003.GIF]
